# Conditioned Media from Microvascular Endothelial Cells Cultured in Simulated Microgravity Inhibit Osteoblast Activity

**DOI:** 10.1155/2014/857934

**Published:** 2014-08-19

**Authors:** Alessandra Cazzaniga, Sara Castiglioni, Jeanette A. M. Maier

**Affiliations:** Dipartimento di Scienze Biomediche e Cliniche Luigi Sacco, Università di Milano, Via GB Grassi 74, Milano, Italy

## Abstract

*Background and Aims*. Gravity contributes to the maintenance of bone integrity. Accordingly, weightlessness conditions during space flight accelerate bone loss and experimental models in real and simulated microgravity show decreased osteoblastic and increased osteoclastic activities. It is well known that the endothelium and bone cells cross-talk and this intercellular communication is vital to regulate bone homeostasis. Because microgravity promotes microvascular endothelial dysfunction, we anticipated that the molecular cross-talk between endothelial cells exposed to simulated microgravity and osteoblasts might be altered. *Results*. We cultured human microvascular endothelial cells in simulated microgravity using the rotating wall vessel device developed by NASA. Endothelial cells in microgravity show growth inhibition and release higher amounts of matrix metalloproteases type 2 and interleukin-6 than controls. Conditioned media collected from microvascular endothelial cells in simulated microgravity were used to culture human osteoblasts and were shown to retard osteoblast proliferation and inhibit their activity. *Discussion*. Microvascular endothelial cells in microgravity are growth retarded and release high amounts of matrix metalloproteases type 2 and interleukin-6, which might play a role in retarding the growth of osteoblasts and impairing their osteogenic activity. *Conclusions*. We demonstrate that since simulated microgravity modulates microvascular endothelial cell function, it indirectly impairs osteoblastic function.

## 1. Introduction

Bone development and remodeling depend mainly upon complex interactions between osteoblasts and osteoclasts. Indeed, an intimate communication exists between osteoblasts and osteoclasts since osteoclasts control osteoblastic growth and function, while osteoblasts regulate the differentiation and the activity of osteoclasts [1]. Recently, other cells of the bone microenvironment are emerging as implicated in bone health. Among others, endothelial cells are players of the communication network in the bone [2]. In embryonic skeletal tissue, osteogenesis and angiogenesis are temporally related [3] and, in the adults, osteoblasts are always located adjacent to endothelial cells in blood vessels at sites of new bone formation [4]. The fact that older subjects with osteoporosis have decreased blood vessels in their skeletal tissue, accompanied by a parallel decrease in osteoblasts, further highlights this close relation [5]. Several lines of evidence indicate that a mutual communication system exists between the endothelium and the osteoblasts. At the cellular and molecular levels, vascular endothelial cells have been shown to regulate bone remodelling via cell signalling networks of ligand-receptor complexes and osteoblasts release growth factors that influence endothelial cells [3].

In long duration space missions, astronauts experience considerable bone loss, about 1-2% of bone mass per month in the weight-bearing regions of the leg and the spine, mainly because of an uncoupling between osteoblasts and osteoclasts [6–8]. We anticipate that endothelial-osteoblast communication might be impaired in space and contributes to bone loss. Indeed, dysfunctions in human endothelial cells cultured in simulated microgravity have been described [9–15], and alterations in the capillaries of the epiphyses and metaphyses of femoral bones of rats flown aboard the US laboratory SLS-2 were detected [16].

Cross-talk between endothelial cells and osteoblasts in simulated microgravity has not been deciphered yet. As a first approach to investigate this issue, we exposed osteoblasts to conditioned media (CM) from microvascular endothelial cells (HMEC) cultured in the rotating wall vessel (RWV), which simulates some aspects of microgravity. Studies utilizing CM are considered a successful strategy for the identification of soluble factors interconnecting different cell types and candidate biomarkers for further validation in clinical samples [17]. Indeed, CM reveal the cell secretome, that is, the collection of proteins that are released through the classical and nonclassical secretion pathways, and also proteins shed from the cell surface. These secreted proteins include enzymes, growth factors, cytokines, and other soluble mediators and are important contributors to cell survival, growth, and differentiation [17]. We here show that CM from HMEC grown in simulated microgravity impair the proliferation and activity of cultured primary osteoblasts and osteoblast-like Saos-2 cells.

## 2. Materials and Methods

### 2.1. Cell Culture

HMEC were obtained from CDC (Atlanta, USA) and grown in MCDB131 containing epidermal growth factor (10 ng/mL) and 10% fetal bovine serum (FBS) on 2% gelatin-coated dishes. Normal human osteoblasts (NHOst) were maintained in osteoblast growth media (OGM) as indicated by the manufacturer (Lonza, Basel, Switzerland) at 37°C in a humidified atmosphere containing 5% CO_2_ [18]. Saos-2 cells (American Type Culture Collection) were cultured in DMEM containing 10% FBS. Before beginning the experiments with CM from HMEC, NHOst and Saos-2 cells were gradually adapted to be cultured in 1 : 1 HMEC growth medium and OGM or DMEM, respectively. To simulate microgravity, we utilized the RWV (Synthecom Inc, Houston, TX, USA). HMEC were seeded on beads (Cytodex 3, Sigma Aldrich, St. Louis, MO, USA); as controls (CTR), HMEC grown on beads were cultured in the vessels not undergoing rotation [11]. In the RWV, the vessel rotates around a horizontal axis (28 rpm) and allows diffusion of oxygen and carbon dioxide across a semipermeable membrane. The vessel wall and the medium containing cells bound to microcarrier beads rotate at the same speed, producing a vector-averaged gravity comparable with that of near-earth free-fall orbit [19]. The beads do not form aggregates in the RWV and tend to be evenly distributed throughout the vessel. Such a rotation reduces gravity to approximately 3 × 10^−2 ^g [10]. After 72 h in the RWV or in the vessels without rotation, the media from HMEC were collected, centrifuged, filtered through 0.2 *μ*m filter, diluted 1 : 1 with fresh culture medium to replenish nutrients, and used to culture NHOst and Saos-2 cells. In these experiments, the medium was changed every 48 h.

### 2.2. DNA Fragmentation

HMEC cell death was evaluated using the cell death detection ELISA (Roche) which determines cytoplasmic histone-associated DNA fragments. Briefly, after 48 and 72 h in the RWV or under control conditions, the cells were lyzed and centrifuged and the supernatant was analyzed according to the manufacturer's instruction. As a positive control, we used HMEC exposed for 30 min to H_2_O_2_ (10 *μ*M) and cultured for additional 48 h in their growth medium.

### 2.3. Cell Proliferation

For MTT assay, NHOst and Saos-2 at 50% confluence were cultured in 96-well plates for 24 h before being exposed for different times to the media collected from HMEC. MTT measures the reduction of yellow tetrazolium salt MTT to dark purple formazan by succinate dehydrogenase, mainly in mitochondria and it is now widely accepted as a reliable way to examine cell viability and proliferation [20]. Briefly, at the end of the experiment, the media were replaced with medium containing 3-(4,5-Dimethyl-2-thiazolyl)-2,5-diphenyl-2H-tetrazolium bromide (MTT, 0.5 mg/mL) (Sigma Aldrich, St. Louis, MO, USA). Formazan crystals generated by the cellular reduction activity were dissolved in DMSO. Absorbance was measured at 550 nm.

Neutral red uptake assay was also used to estimate NHOst viability. Briefly, 24 h after seeding in 96-well dishes, the cells were exposed to CM from HMEC. After 3 days, neutral red was added to the medium to a final concentration of 50 *μ*g/mL. 2 h later, the wells were washed with PBS and fixed. Absorbance was measured at 550 nm [21].

HMEC and Saos-2 cells were trypsinized and stained with trypan blue solution (0.4%) and the viable cells were counted using a Burker chamber.

### 2.4. Osteoblast Activity

NHOst and Saos-2 cells at 80% confluence were cultured in 24-well plates with conditioned media from HMEC added with 100 nM dexamethasone, 50 *μ*M L-ascorbate-2-phosphate, and 10 mM glycerophosphate, at 37°C in a 5% CO_2_ for 7 and 14 days. Osteoblast activity was evaluated quantifying alkaline phosphatase (ALP) enzymatic activity in the medium by a colorimetric assay based on the hydrolysis of P-nitrophenyl phosphate. The absorbance was measured at 405 nm [18]. To analyze calcium deposition, the cells were rinsed with PBS, fixed (70% ethanol, 1 h), and stained for 10 min with 2% Alizarin Red S (pH 4.2). Cultures were photographed with a digital camera. Alizarin Red was then released from the cell matrix by incubation for 15 min in 10% cetylpyridinium chloride in 10 mM sodium phosphate (pH 7.0). The absorbance was measured at 562 nm [18].

### 2.5. Measurements of TIMP-2 and IL-6 by ELISA

Conditioned media were centrifuged and filtered. The amounts of tissue inhibitor of matrix metalloprotease (TIMP)-2 and interleukin (IL)-6 were measured using a double-antibody sandwich ELISA (GE Healthcare) according to the manufacturer's instructions. The concentrations of TIMP-2 and IL-6 were determined by interpolation from a standard curve.

### 2.6. Western Blot

HMEC cells were lysed, separated on SDS-PAGE, and transferred to nitrocellulose sheets. Western analysis was performed using antibodies against p21, p53, and GAPDH (Tebu Bio-Santa Cruz). Secondary antibodies were labelled with horseradish peroxidase (Amersham Pharmacia Biotech). The SuperSignal chemiluminescence kit (Pierce) was used to detect immunoreactive proteins.

### 2.7. Statistical Analysis

All experiments were repeated at least three times in triplicate. Data are presented as means ± standard deviation. Statistical differences were determined using the unpaired two-tailed Student's *t* test. Consider *P < 0.05, **P < 0.01.

## 3. Results

### 3.1. Simulated Microgravity Alters HMEC Behaviour

**Figure 1:**
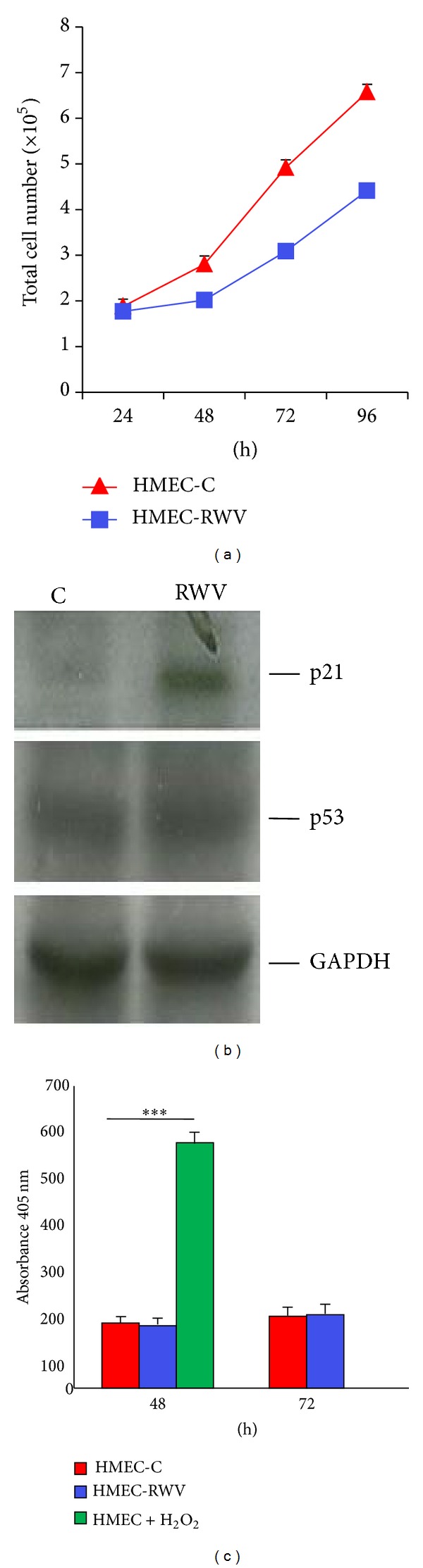
Simulated microgravity inhibits HMEC growth. (a) HMEC were cultured for different times in the RWV (HMEC-RWV) and trypsinized and viable cells were counted. HMEC-C: control. (b) Cell extracts (50 *μ*g/lane) were loaded on a 15% SDS-PAGE, blotted into nitrocellulose filter, incubated with anti-p21 and anti-p53 antibodies, and visualized by chemiluminescence as described. After stripping, the blot was incubated with an anti-GAPDH antibody to show that comparable amounts of proteins were loaded per lane. (c) Apoptosis was evaluated by ELISA on HMEC lysates after 48 and 72 h in the RWV or under control conditions. Our positive control is represented by HMEC exposed to H_2_O_2_ for 30 min and then cultured for additional 48 h.

**Figure 2:**
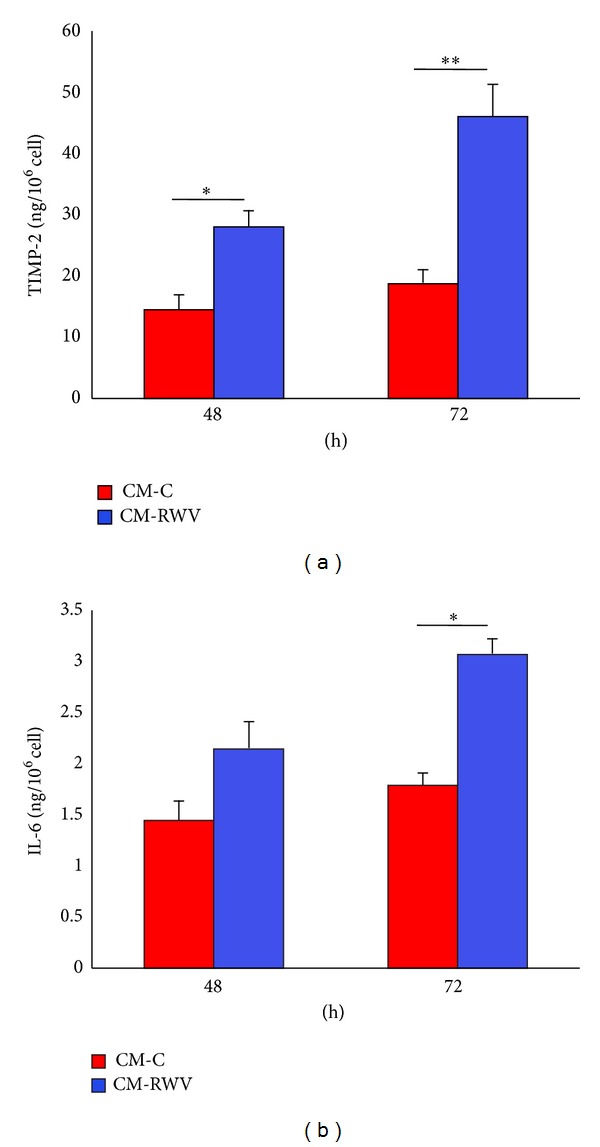
Simulated microgravity induces TIMP-2 and IL-6 secretion by HMEC. TIMP-2 (a) and IL-6 (b) were measured by ELISA in media collected after different times of culture in the RWV (CM-RWV) or from relative controls (CM-C).

Figure 1(a) shows that culture in the RWV retarded HMEC proliferation. Accordingly, growth inhibition correlated with the upregulation of p21 (WAF1), an inhibitor of cyclin-dependent kinases, as detected by western blot, and this event seems to be p53-independent since no modulation of p53 was observed in HMEC (Figure 1(b)). We also show that no cell death is detectable after 48 and 72 h culture in the RWV (Figure 1(c)). It is noteworthy that similar results were obtained when microgravity was simulated using the random positioning machine (RPM) (data not shown). On the basis of results obtained by protein array on 40 proteins involved in inflammation, we validated the increase of IL-6 and TIMP-2 in the CM from HMEC cultured for 48 and 72 h in the RWV and relative controls by ELISA.  Figure 2(a) shows that TIMP-2 is significantly increased in the media collected from HMEC after 48 and 72 h in the RWV, while secreted IL-6 was increased after 72 h culture in simulated microgravity (Figure 2(b)). On these bases, we decided to use 72 h conditioned media from HMEC for the experiments on bone cells.

### 3.2. HMEC Secreted Factors Impact on NHOst Cell Proliferation and Osteogenic Activity

**Figure 3:**
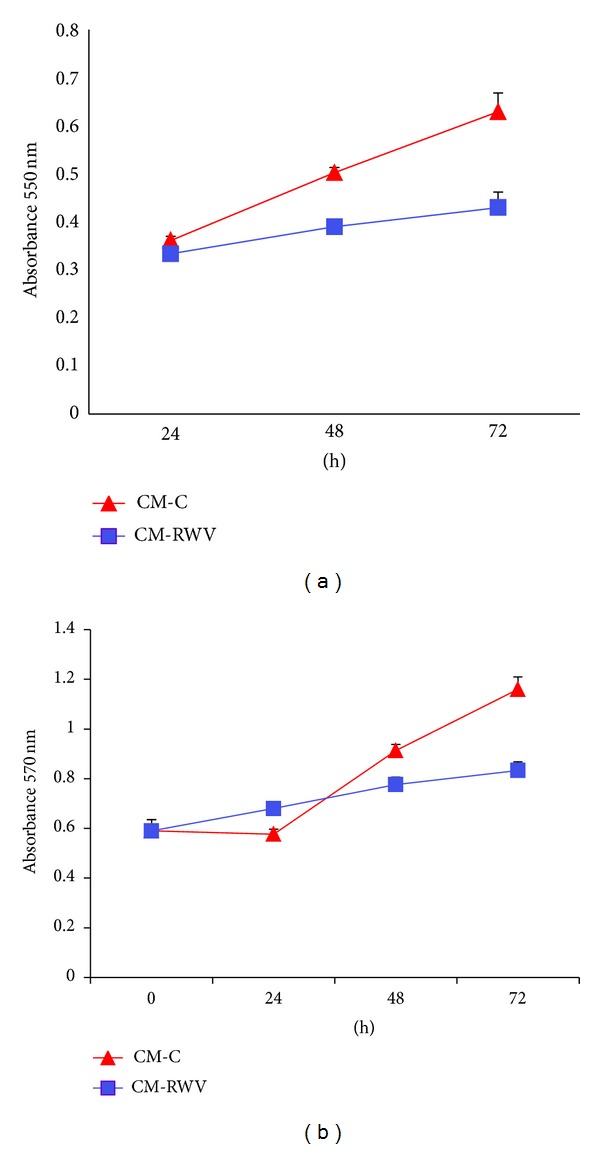
CM from HMEC in simulated microgravity inhibit NHOst proliferation. NHOst were cultured for different times with CM from HMEC in simulated microgravity (CM-RWV) or by HMEC controls (CM-C). Viable cells were evaluated by MTT assay (a) and neutral red (b) and the absolute absorbance values are shown. Data are expressed as the mean ± standard deviation of three different experiments performed in triplicate.

We evaluated the effects of CM from HMEC on NHOst cell proliferation. MTT assay revealed a significant reduction of NHOst cell proliferation cultured in the presence of CM from HMEC in simulated microgravity (Figure 3(a)). These results were confirmed by neutral red assay, which estimates the number of viable cells in a culture on the basis of their ability to incorporate and bind the supravital dye neutral red in the lysosomes (Figure 3(b)). We did not detect any significant difference in cell death in NHOst exposed to the conditioned media from HMEC cultured for 72 h in the RWV and relative controls (not shown).

To evaluate osteoblastic activity, NHOst cells were cultured for 7 and 14 days in a 24-well plate with CM from HMEC added with an osteogenic cocktail containing 100 nM dexamethasone, 50 *μ*M L-ascorbate-2-phosphate, and 10 mM glycerophosphate. Two parameters were evaluated, that is, ALP activity, which has long been recognised as a reliable indicator of osteoblastic activity, and calcium deposition by Alizarin Red Staining.

**Figure 4:**
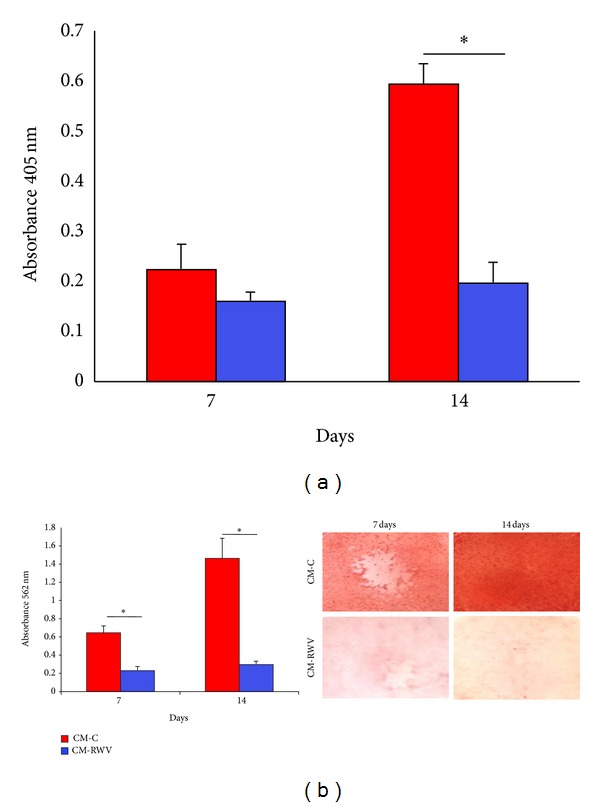
CM from HMEC in simulated microgravity inhibit NHOst activity. NHOst were cultured for 7 and 14 days with medium conditioned by HMEC in simulated microgravity (CM-RWV) or by HMEC controls (CM-C) both added with osteogenic stimuli. (a) ALP enzymatic activity was quantified by spectrophotometric analysis as described. Absorbance was measured at 405 nm. (b) Alizarin Red Staining was performed. Photographs were taken before acid extraction. Absorbance was measured at 562 nm.

ALP enzymatic activity was measured after 7 and 14 days by a colorimetric assay.  Figure 4(a) shows that media from HMEC in simulated microgravity inhibited ALP activity. To analyze calcium deposition, we used the Alizarin Red S Staining.  Figure 4(b) shows that CM from HMEC exposed to simulated microgravity markedly inhibited the deposition of mineral matrix.

### 3.3. HMEC Secreted Factors Impact on Saos-2 Cell Proliferation and Osteogenic Activity

Many factors, such as age, gender, and site of isolation, influence the behavior of primary osteoblasts [22]. We therefore performed experiments also on an immortalized cell line to reproduce the results obtained in NHOst and we chose Saos-2 cells because they closely resemble primary osteoblasts [22]. Indeed, Saos-2 cells are used as representative of primary osteoblasts when standard tests are evaluated [23].

**Figure 5:**
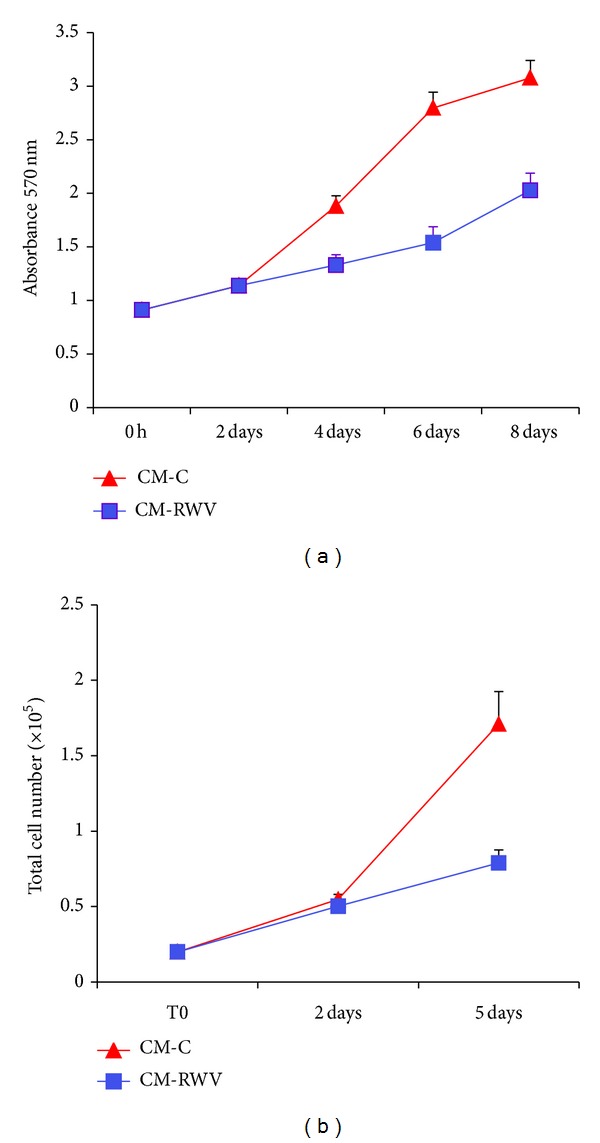
CM from HMEC in simulated microgravity inhibit Saos-2 proliferation. Saos-2 were cultured for different times with CM from HMEC in simulated microgravity (CM-RWV) or by HMEC controls (CM-C). Viable cells were evaluated by MTT assay (a) and the absolute absorbance values are shown. After trypsinization, viable cells were stained with trypan blue and counted (b).

Saos-2 cells were exposed to CM from HMEC in the RWV and relative controls for different times. MTT assay shows that media from HMEC in the RWV impair cell proliferation (Figure 5(a)). These results were confirmed when the cells were counted (Figure 5(b)).

**Figure 6:**
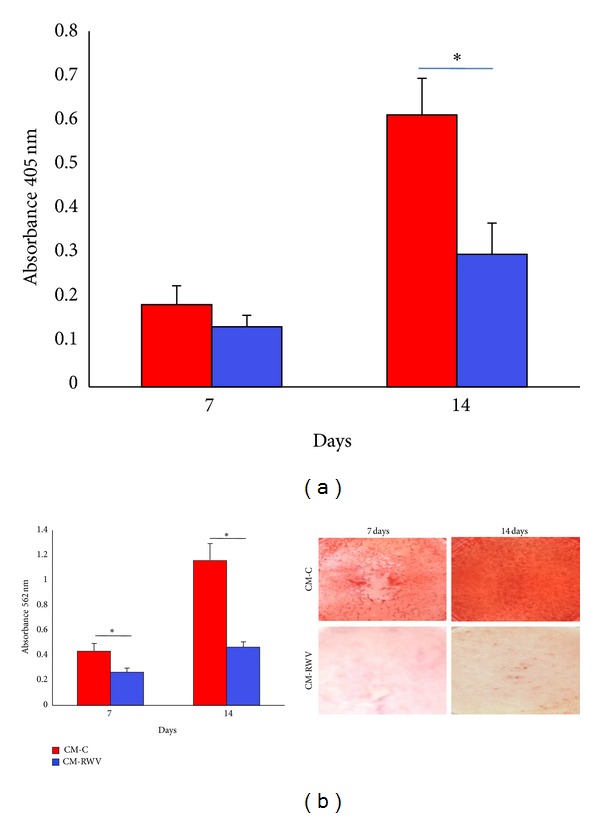
CM from HMEC in simulated microgravity inhibit Saos-2 activity. Saos-2 were cultured for 7 and 14 days with CM from HMEC in simulated microgravity (CM-RWV) or by HMEC controls (CM-C) both added with osteogenic stimuli. (a) ALP enzymatic activity and (b) Alizarin Red Staining were performed as above.

Confluent Saos-2 cells were then cultured in CM from HMEC in simulated microgravity or HMEC controls both added with the osteogenic cocktail and were stained with Alizarin Red to evaluate the formation of calcium phosphate in culture [18]. We found that 14-day culture in the conditioned media from HMEC in the RWV inhibited ALP activity (Figure 6(a)). The inhibition of Saos2 cell activity was confirmed by demonstrating lower amounts of deposition of mineral matrix in cell cultured with the CM from HMEC in the RWV (Figure 6(b)).

## 4. Discussion

Bone loss in space has been reported in humans and in several experimental models [8]. All the* in vivo* results obtained in space point to major alterations of bone cells. Bone cells have been extensively studied* in vitro* both in space and on ground using different devices to simulate microgravity to conclude that microgravity alters the morphology of these cells [24], impairs the differentiation of osteoblasts [25], and increases the activity of osteoclasts [8]. All these results are not surprising since gravitational forces contribute to the maintenance of bone integrity and affect bone remodeling to adjust to mechanical demands.

Bone vasculature is important for skeletal development during the embryonic stage, postnatal growth, and bone remodeling. It supplies oxygen, nutrients, hormones, cytokines, and bone precursor cells. Moreover, the communication between bone endothelium and bone cells is vital to regulate and modulate bone homeostasis. The endothelium contributes to bone health by releasing osteogenic factors [26], and bone cells produce angiogenic factors that are crucial for endothelial viability and survival under physiological conditions and that drive angiogenesis when needed [3].

We have shown that human endothelial cells from the umbilical vein, widely used as a model of macrovascular endothelial cells, are deeply influenced by simulated microgravity [10, 11, 27]. These results were confirmed by our recent study performed on the International Space Station (ISS) [28]. Other experiments have been performed on different types of macrovascular endothelial cells with discordant results, which can be ascribed to poor definition of the endothelial cells used [14, 15], the different culture conditions, the use of different microgravity simulators, and also the inadequate descriptions of how they were operated. Less is known about microvascular endothelial cells, which cover an area 50 times greater than that of all large vessels combined [29]. In an animal model of wound healing and in a rat fibular osteotomy model, microgravity retards neovascularization [30, 31], thus indicating the occurrence of microvascular endothelial dysfunction. Moreover, bed rest, which mimics some aspects of spaceflight, causes impairment of endothelium-dependent functions in the microcirculation [32]. We have previously demonstrated that RWV-simulated microgravity induces an antiangiogenic phenotype in HMEC [11]. In the present study, we confirm and broaden these results by showing that culture in the RWV retards HMEC cell growth without inducing apoptosis. This correlates with the upregulation of p21, an inhibitor of the cyclin/CDK2 complexes necessary for the transition from the G1 to the S phases, through a p53-independent mechanism. Our results are in disagreement with a recent report showing that culture in a clinostat induces apoptosis in pulmonary microvascular endothelial cells [12]. As mentioned above, these contrasting results might be due to differences in the cells used, in the cell culture conditions, and in the microgravity simulator utilized.

The aim of this work was to understand whether simulated microgravity impairs endothelial-osteoblast communication. To this purpose, we evaluated the effects produced on osteoblasts by CM from HMEC cultured in simulated microgravity.

We show that HMEC release factors that retard the growth of osteoblasts and severely impair their osteogenic activity. It is noteworthy that we found increased amounts of secreted TIMP-2 and IL-6, known to affect both endothelial cells and osteoblasts. Interestingly, TIMP-2 inhibits endothelial cell proliferation by a matrix metalloproteases (MMP) independent mechanism [33] and might therefore play a role in HMEC growth retardation in simulated microgravity. TIMP-2 also impairs osteoblast activity. Indeed, TIMP-2 nearly abolishes ALP expression [34] by inhibiting MT1-MMP (membrane type 1-metalloprotease) [34], a protease which is implicated in multiple steps of osteogenic differentiation and is mainly involved in ALP upregulation [35]. Interestingly, TIMP-2 inhibits cell survival of osteoblasts forced to transdifferentiate into osteocytes [36]. This result might offer a molecular explanation, at least in part, to the lysis of osteocytes in spaceflight described by Blaber et al. [37]. In media from HMEC cultured in the RWV, we also found increased amounts of IL-6, a pleiotropic cytokine implicated in acute phase response and inflammation. IL-6 not only promotes endothelial dysfunction [38] but also affects human osteoblast differentiation [39], thus contributing to osteopenia.

We therefore propose that microgravity impacts both directly and indirectly on osteoblasts. Microgravity has been shown to directly inhibit osteoblasts. In addition, by modulating microvascular endothelial cell function, microgravity indirectly exerts inhibitory effects on osteoblasts.

The current space programs onboard the ISS and the future human exploration of Mars require long duration missions. However, several biomedical issues still need to be clarified before these missions can take place without causing health problems to the astronauts. Our results suggest that endothelial dysfunction might represent a common denominator for cardiovascular deconditioning and for bone loss and offer a new light to interpret the behaviour of mammalian skeleton in microgravity. Eventually, these results might foster studies to develop countermeasures that target the endothelium to improve both bone homeostasis and vascular function.
